# Surface modification processes during methane decomposition on Cu-promoted Ni–ZrO_2_ catalysts

**DOI:** 10.1039/c4cy00988f

**Published:** 2014-10-27

**Authors:** Astrid Wolfbeisser, Bernhard Klötzer, Lukas Mayr, Raffael Rameshan, Dmitry Zemlyanov, Johannes Bernardi, Karin Föttinger, Günther Rupprechter

**Affiliations:** a Institute of Materials Chemistry , Vienna University of Technology , Getreidemarkt 9 , 1020 Wien , Austria . Email: karin.foettinger@tuwien.ac.at ; Fax: +43 1 58801 16599 ; Tel: +43 1 165110; b Institute of Physical Chemistry , University of Innsbruck , Innrain 52a , 6020 Innsbruck , Austria; c Purdue University , Brick Nanotechnology Center , 1205 West State Street , West Lafayette , IN 47907-2057 , USA; d University Service Center for Transmission Electron Microscopy , Vienna University of Technology , Wiedner Hauptstraße 8-10 , 1040 Wien , Austria

## Abstract

The surface chemistry of methane on Ni–ZrO_2_ and bimetallic CuNi–ZrO_2_ catalysts and the stability of the CuNi alloy under reaction conditions of methane decomposition were investigated by combining reactivity measurements and *in situ* synchrotron-based near-ambient pressure XPS. Cu was selected as an exemplary promoter for modifying the reactivity of Ni and enhancing the resistance against coke formation. We observed an activation process occurring in methane between 650 and 735 K with the exact temperature depending on the composition which resulted in an irreversible modification of the catalytic performance of the bimetallic catalysts towards a Ni-like behaviour. The sudden increase in catalytic activity could be explained by an increase in the concentration of reduced Ni atoms at the catalyst surface in the active state, likely as a consequence of the interaction with methane. Cu addition to Ni improved the desired resistance against carbon deposition by lowering the amount of coke formed. As a key conclusion, the CuNi alloy shows limited stability under relevant reaction conditions. This system is stable only in a limited range of temperature up to ~700 K in methane. Beyond this temperature, segregation of Ni species causes a fast increase in methane decomposition rate. In view of the applicability of this system, a detailed understanding of the stability and surface composition of the bimetallic phases present and the influence of the Cu promoter on the surface chemistry under relevant reaction conditions are essential.

## Abstract 2

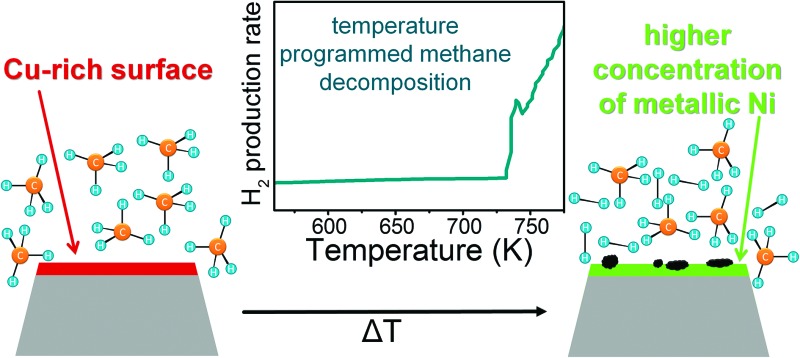
We explored the surface chemistry of methane on Cu-promoted Ni–ZrO_2_ catalysts and observed a limited stability of the CuNi alloy under relevant reaction conditions.

## Introduction

Hydrogen is known as a promising source of energy and fuel. Up to now hydrogen is mostly produced out of hydrocarbons such as methane. *Via* steam reforming^1–4^ or partial oxidation^5,6^ of methane, CO or CO_2_ is formed as a by-product. For many applications such as the use of hydrogen in PEM fuel cells CO needs to be removed because it is a catalyst poison. One possibility to overcome the problem of CO or CO_2_ formation is the direct production of hydrogen out of methane by catalytic decomposition in the absence of air or oxygen. The by-product of this process is carbon in the form of layers, filaments, fibres or nanotubes. Especially, the formation of useful nanocarbons^7–14^ is of high interest. Li *et al.* ^15^ published a comprehensive review about this topic discussing the effect of the nature of the catalyst and feed composition on the catalytic activity, stability and the influence on the morphology of the produced carbon.

During methane decomposition, methane is adsorbed on the catalyst followed by C–H bond breaking, deposition of carbon and generation of hydrogen. Transition metals such as Ni,^16–18^ Fe^17^ and Co^19^ are known to be effective for methane decomposition to hydrogen and carbon nanofibres. Ni catalysts are known for their high activity but are easily deactivated due to carbon deposition. However, nickel-based catalysts have been proven to be sensitive to doping and structure modification by other metals, such as Cu, which affects the structure and morphology of the produced carbon.^8^ Improved stability against deactivation has been achieved by adding a second metal component; for example, Au^20–23^ and Cu^13,24–32^ have been studied for this aim. The Ni–Au surface alloy was active for steam reforming and more resistant toward carbon formation than the pure Ni, which was explained by blocking of highly reactive Ni edge and kink sites by Au atoms.^21^ The addition of Cu to nickel-based catalysts has several effects such as an easier reducibility of Ni. Cu is rich in d-electrons which may exert an electronic effect on Ni and furthermore affect the affinity of the metal surface to graphite.^15^ Ashok *et al.* observed that addition of Cu to Ni enhanced the methane decomposition activity of hydrotalcite-like Ni–Cu–Al catalysts when added in an appropriate amount.^26^ Suelves *et al.* showed that doping of Cu has a strong influence on the dispersion of nickel and enhances the catalyst activity.^30^ Similarly, Monzón found that promoting Ni with a small amount of Cu improves the activity and stability of the catalyst.^31^ Cunha *et al.* reported that doping with Cu improves the catalyst stability.^32^ Li *et al.* observed that in Cu_0.25_Ni_0.75_Al_2_O_4_ Cu is acting as a dispersive agent to prevent Ni from growing^24^ and enhances the catalyst stability when employed in a suitable amount.^13^ Lazaro *et al.* found that the presence of copper in NiCu–SiO_2_ catalysts led to higher methane conversion,^28^ and Park *et al.* observed that on Cu–Ni–YSZ anode materials for the direct use of methane fuel in solid oxide fuel cells (SOFC) carbon deposition decreased compared to the amount of carbon on Ni–YSZ.^29^ DFT-based computational studies by Liu *et al.* compared methane dissociation on NiCu(111) bimetallic alloy surfaces. Their calculations indicated that C is easily formed on a uniform 1 : 1 NiCu surface but unfavourable on a Cu-rich NiCu surface.^33^ Therefore, the surface composition on a NiCu alloy catalyst under reaction conditions is expected to play the key role in determining the catalytic activity and stability.

One prerequisite for a successful promoter is *in situ* stability under relevant reaction conditions, *i.e.* high temperatures and reactive surrounding gas atmosphere. Therefore, understanding the stability of the bimetallic phases and the influence of the promoter on the surface chemistry are essential. We have studied CH_4_- and methanol reforming activities and selectivities on Ni-, Cu- and Pd-based catalyst systems by combining *in situ* spectroscopic techniques such as XPS, FTIR and XAS.^25,34–39^ In the present work, we have selected Cu as an exemplary compound for modifying the reactivity of Ni in methane decomposition and enhancing the resistance against coke formation.

In this work we focus on the surface chemistry of methane on Ni–ZrO_2_ catalysts during methane decomposition at temperatures up to 773 K and study the stability of the CuNi alloy under reaction conditions. By *in situ* synchrotron-based near-ambient pressure (NAP-)XPS we determined the surface composition of the catalysts and the nature of the carbonaceous species present and correlated these data with the catalytic performance. Cu addition to Ni improved the desired reduction of carbon deposition,^40^ but only in a limited range of temperature due to the limited stability of this system.

## Experimental

### Sample preparation

Ni–ZrO_2_ and bimetallic CuNi–ZrO_2_ catalysts were synthesized by impregnation of ZrO_2_ using nitrate precursor salts. Prior to impregnation, commercial Zr(OH)_4_ (MEL Chemicals XZO 880/01) was calcined at a heating rate of 2 K min^–1^ from room temperature to 973 K and kept at this temperature for 2 h. After calcination the zirconia support exhibits a specific surface area of 36.6 m^2^ g^–1^.

Bimetallic CuNi samples were prepared by coimpregnation. Cu(NO_3)2_·3H_2_O (Fluka, p.a.) and Ni(NO_3_)_2_·6H_2_O (Merck, p.a.) were mixed to obtain the following Cu : Ni molar ratios: 1 : 0, 3 : 1, 1 : 1, 1 : 3 and 0 : 1. The nitrates were dissolved in water, and in each case 4.75 g of ZrO_2_ powder was suspended in these solutions. Every solution contained as much metal nitrate to obtain 5 wt% metal loading in the final catalyst powder. Additionally, a 5 wt% Cu–ZrO_2_ reference material was synthesized by the same procedure.

For *in situ* X-ray photoelectron spectroscopy (XPS) investigations an additional sample was prepared *via* the same synthesis route with a higher overall metal loading of 50 wt% in order to prevent sample charging.

All catalysts and their respective compositions are summarized in Table 1.

**Table 1:** Composition of CuNi–ZrO_2_ catalysts

Sample name	ZrO_2_ (wt%)	Cu (wt%)	Ni (wt%)
Ni–ZrO_2_	95	—	5
13CuNi–ZrO_2_	95	1.25	3.75
11CuNi–ZrO_2_	95	2.5	2.5
31CuNi–ZrO_2_	95	3.75	1.25
Cu–ZrO_2_	95	5	—
13CuNi–ZrO_2_50	50	12.5	37.5

### Characterization

Transmission electron microscopy (TEM) was utilized to obtain information about the size, morphology and distribution of the metal particles on the zirconia support. For TEM measurements the samples were deposited on carbon-coated Cu grids (Ni–ZrO_2_) or Au grids (all copper-containing samples). The catalysts were oxidized at 773 K and reduced at 673 K prior to the experiment. TEM measurements were performed using an analytical TECNAI F20 instrument connected to an EDX detector. EDX was used to identify Cu, Ni or bimetallic CuNi particles. For the bimetallic catalyst elemental mapping was additionally carried out.

### Reactivity for methane decomposition

Temperature-programmed methane decomposition (TPMd) experiments were performed in a quartz flow reactor. Part of the effluent was directed to a mass spectrometer (Pfeiffer QMS 200) *via* a differentially pumped capillary. Thereby the main products (H_2_, CO, CO_2_ and CH_4_) were detected. 50 mg of the catalyst was loaded into the glass reactor in-between two plugs of quartz wool. The catalysts were oxidized at 773 K and reduced at 673 K prior to the reaction. Then the samples were heated up to 773 K and cooled down to 573 K in a mixture of 5% CH_4_ and 95% Ar with a total flow of 50 ml min^–1^. The heating and cooling rate was set to 5 K min^–1^ and the heating–cooling cycle was repeated thrice. After the TPMd experiment the sample was cooled down to room temperature in Ar. Subsequently, temperature-programmed oxidation (TPO) was performed in 20% O_2_ in Ar applying a heating rate of 5 K min^–1^ up to 673 K.

For the quantitative determination of the reaction rates the reaction products were analyzed under steady-state conditions by a gas chromatograph equipped with a thermal conductivity detector (TCD) and flame ionization detector (FID).

### *In situ* XPS measurements

*In situ* synchrotron-based near-ambient pressure (NAP-)XPS measurements were carried out at the ISSIS-PGM beamline at the Helmholtz-Zentrum Berlin using a high-pressure XPS station constructed at the FHI Berlin. Details of the setup are described elsewhere.^41^ Briefly, the setup uses a differentially pumped electrostatic lens system and a SPECS hemispherical electron analyser.

The sample was placed inside a reaction cell with adjustable gas flow and a total pressure up to the millibar range. The composition of the gas phase was analysed by MS. Heating was done by an IR laser from the back side. The sample was *in situ* oxidized in 0.1 mbar O_2_ at 573 K and reduced in 0.25 mbar H_2_ at 673 K prior to exposure to 0.25 mbar CH_4_ at temperatures ranging from 523 up to 723 K.

The catalyst 13CuNi–ZrO_2_50 with 50 wt% total metal loading was used for these experiments which prevented sample charging. The calcined powder was pressed onto a copper plate and reduced in 0.25 mbar H_2_ at 773 K prior to placing it into the *in situ* reaction cell. Measurements were performed at photon energies of 1100 eV for the Cu 2p, 1010 eV for the Ni 2p and 425 eV for the C 1s XP spectra, yielding a photoelectron kinetic energy of 150 eV. For depth profiling the photon energies were varied to obtain additional photoelectron kinetic energies of 350 eV and 550 eV. All spectra were corrected for synchrotron beam current, incident photon flux and energy-dependent photo-ionization cross sections.^42^ Binding energies (BEs) were referenced to the Fermi edge recorded after each core-level measurement. The measured signal envelopes were fitted to Gauss–Lorentzian peaks by using Casa XPS software after subtraction of a Shirley background.

## Results

### Transmission electron microscopy

**Fig. 1:**
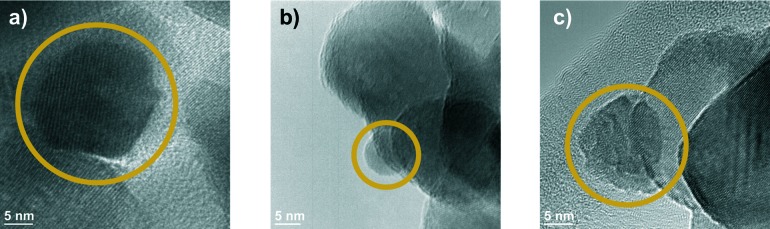
TEM images of a) Ni–ZrO_2_, b) Cu–ZrO_2_ and c) 11CuNi–ZrO_2_. The particles inside the circles were identified *via* EDX as Ni in a), Cu in b) and CuNi in c).

Fig. 1 shows transmission electron micrographs of Ni–ZrO_2_, Cu–ZrO_2_ and 11CuNi–ZrO_2_ after *ex situ* reduction.

**Fig. 2:**
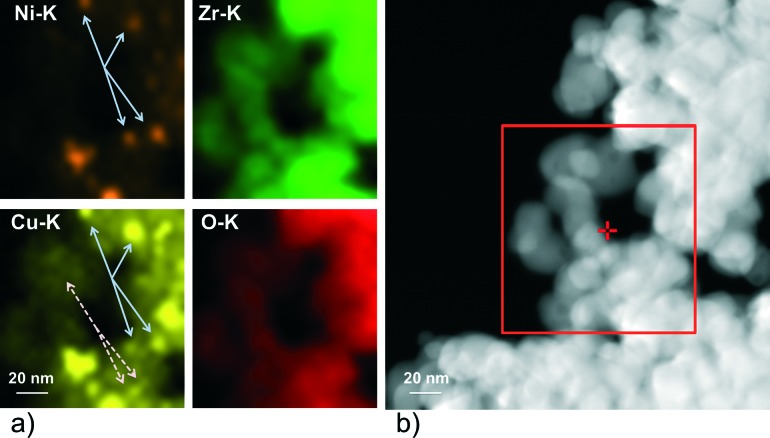
a) Elemental map of the region shown in the rectangle in b) for the K edge energies of Ni, Cu, Zr and O recorded on 11CuNi–ZrO_2_. Full arrows: particles containing both Ni and Cu, dashed arrows: Cu but no Ni detected.

The size of the ZrO_2_ particles was between 50 and 100 nm. In general the metal particles were found to be evenly distributed on the oxide support. The Ni particles in Ni–ZrO_2_ exhibit a homogeneous size distribution of approximately 20 nm mean size and are of polyhedral shape. The Cu particles in Cu–ZrO_2_ are spherical and about 5 nm in size. In contrast, the metal particles on the bimetallic 11CuNi–ZrO_2_ with average sizes of around 20 nm are polycrystalline and rather irregular in shape. EDX spectra showed that those particles contained both Cu and Ni, although not in the expected 1 : 1 ratio, but they were enriched in nickel. To get a better understanding of the Cu and Ni distribution on the bimetallic catalyst, elemental maps were recorded. The results are displayed in Fig. 2.

While Ni was always accompanied by the simultaneous presence of Cu as demonstrated with full arrows in Fig. 2, Cu was additionally found finely distributed in regions where no or hardly any nickel was detected (dashed arrows in Fig. 2). Thus, the elemental maps revealed the presence of Ni-rich bimetallic particles as well as monometallic Cu particles. XANES spectra of the same catalyst during reduction confirmed the presence of bimetallic CuNi particles.^25^ The observation that the presence of copper improves the reducibility of nickel and lowers its reduction temperature is in agreement with TPR experiments reported in the literature.^9,26,27,43–45^

### Temperature-programmed methane decomposition

**Fig. 3:**
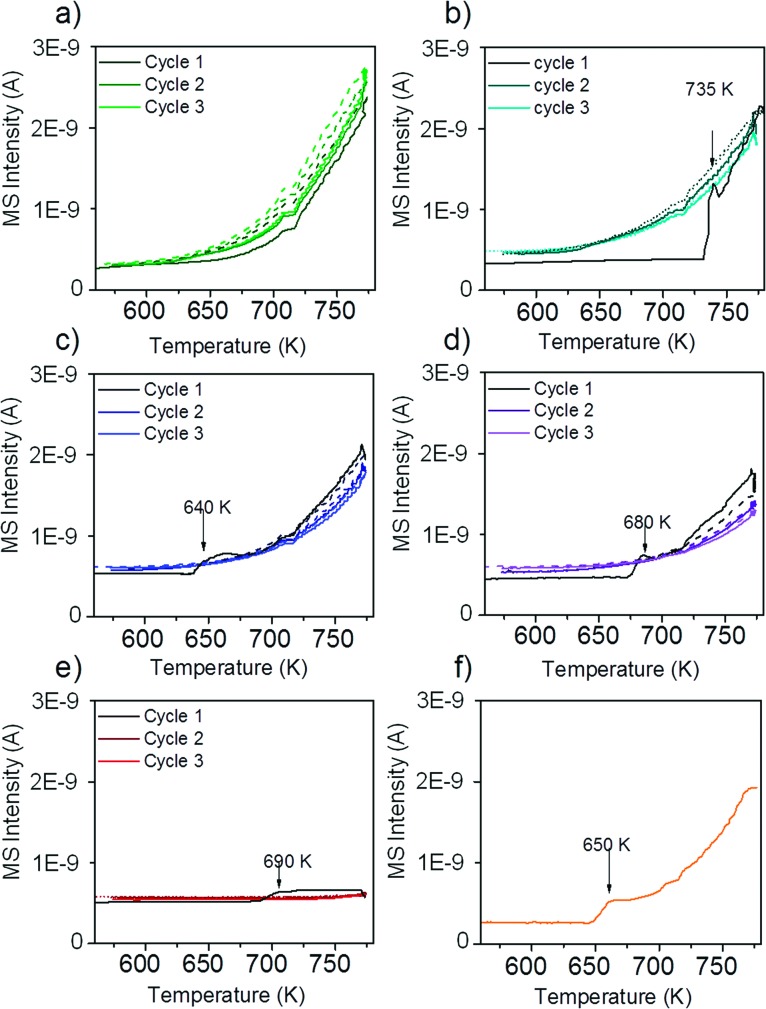
Hydrogen evolution during methane TPMd on a) Ni–ZrO_2_, b) 13CuNi–ZrO_2_, c) 11CuNi–ZrO_2_, d) 31CuNi–ZrO_2_, e) Cu–ZrO_2_ and f) 13CuNi–ZrO_2_50. The samples a)–f) were reduced at 673 K followed by 3 heating–cooling cycles between 573 and 773 K in methane/Ar. For f) only one heating cycle is shown. The temperature was ramped at 5 K min^–1^.

The reactivity of the catalysts for decomposition of methane was investigated by TPMd measurements. The results obtained over Ni–ZrO_2_ are shown in Fig. 3a).

The production of hydrogen started at around 625 K, increased further with increasing temperature and decreased again reversibly when the sample was cooled down to 573 K. Upon heating again to 773 K, the hydrogen production rate remained approximately the same as in the first cycle. Thus, no deactivation of H_2_ production activity was observed. Besides H_2_ formation, carbon dioxide evolution occurred during the first heating cycle in methane up to ~700 K. In the second and third heating cycles a small CO_2_ desorption peak was observed with a maximum at 630 K. The detected CO_2_ likely originates from the reaction of methane with surface oxygen and carbonates of zirconia or with NiO present due to incomplete reduction.

Fig. 3b) shows the rate of hydrogen production over 13CuNi–ZrO_2_ under the same conditions. Over this sample no catalytic activity was found up to 735 K. At this temperature, the amount of produced hydrogen increased rapidly up to a level similar to that over Ni–ZrO_2_. The onset of H_2_ production was accompanied by a small amount of CO_2_ evolving which occurred only in the first heating cycle. During the second and third reaction cycles about the same amount of hydrogen was detected than that over Ni–ZrO_2_. This indicates an irreversible surface modification process on the bimetallic 13CuNi–ZrO_2_ sample occurring during the first heating cycle in methane which significantly changed the catalytic performance of the catalyst.

On 11CuNi–ZrO_2_ and 31CuNi–ZrO_2_ as well as 13CuNi–ZrO_2_50, shown in Fig. 3c), d) and f), a behaviour was observed similar to that on 13CuNi–ZrO_2_ but the activation step occurred at 640 K, 680 K and 650 K, respectively, and the activity for H_2_ formation was lower than that on the Ni-rich sample. Over Cu–ZrO_2_, shown in Fig. 3e), hardly any hydrogen production was observed. During the first heating cycle a small amount of hydrogen was produced above 690 K but decreased again during the isothermal period at 773 K. A small amount of CO_2_ was also detected during the first heating cycle. Afterwards, Cu–ZrO_2_ was completely inactive. The low initial activity can likely be attributed to the reaction of methane with surface oxygen and hydroxide species from zirconia or incompletely reduced Cu species and are not available anymore in the second and third reaction cycles. Often CO is reported as a by-product of the reaction between methane and oxygen species on the support or the incompletely reduced metal oxides.^46,47^ However, formation of various surface carbonate species was observed from CO on zirconia and all zirconia-supported catalysts by IR spectroscopy, which likely decompose to CO_2_.

In order to find out whether hydrogen evolution occurs because of further reduction of the catalyst in methane, the measurement was repeated with the same sample but after reduction at 773 K instead of 673 K. The sudden onset in hydrogen formation accompanied by carbon dioxide evolution was also observed, but the onset temperature was lowered to 650 K.

For quantitative comparison of the catalytic activity the amount of produced hydrogen was determined by GC-TCD.

Table 2 shows the hydrogen yields and reaction rates for hydrogen production during steady-state methane decomposition at 773 K.

**Table 2:** Hydrogen yields and initial reaction rates during methane decomposition at 773 K in 5% CH_4_ and 95% Ar with a total flow of 50 ml min^–1^

Sample	H_2_ yield	Reaction rate *r* per g catalyst (mol H_2_/(g_catalyst_ s^–1^)	Reaction rate *R* per g nickel (mol H_2_/(g_nickel_ s^–1^)
Ni–ZrO_2_	63.3%	2.4 × 10^–5^	4.8 × 10^–4^
13CuNi–ZrO_2_	49.8%	1.9 × 10^–5^	5.0 × 10^–4^
11CuNi–ZrO_2_	16.9%	6.4 × 10^–6^	2.6 × 10^–4^
31CuNi–ZrO_2_	13.6%	5.1 × 10^–6^	4.1 × 10^–4^
Cu–ZrO_2_	1.5%	5.6 × 10^–7^	—
13CuNi–ZrO_2_50	40.0%.	1.5 × 10^–5^	3.9 × 10^–5^

The initial reaction rate *r* calculated per gram catalyst decreased with decreasing overall Ni content. However, the rate *R* normalized per gram Ni is of about the same order of magnitude for Ni and all bimetallic samples containing 5 wt% total loading. In agreement with TPMd measurements, similar rates of hydrogen formation were detected on Ni-rich 13CuNi–ZrO_2_ compared to those on Ni–ZrO_2_. Cu is almost inactive and the reaction rate decreases fast to zero with time on-stream while it stays constant for the other catalysts for at least 1 hour. The rate *r* of the catalyst containing 50 wt% metals, 13CuNi–ZrO_2_50, is about the same as that of 13CuNi–ZrO_2_ with 5 wt% metal loading, which results in a reaction rate *R* per gram nickel of one order of magnitude lower. This can probably be explained by a lower Ni dispersion at such high loading. In general, we did not observe an enhancement of the catalytic activity by addition of copper to the Ni–ZrO_2_ catalyst.

### Temperature-programmed oxidation

**Fig. 4:**
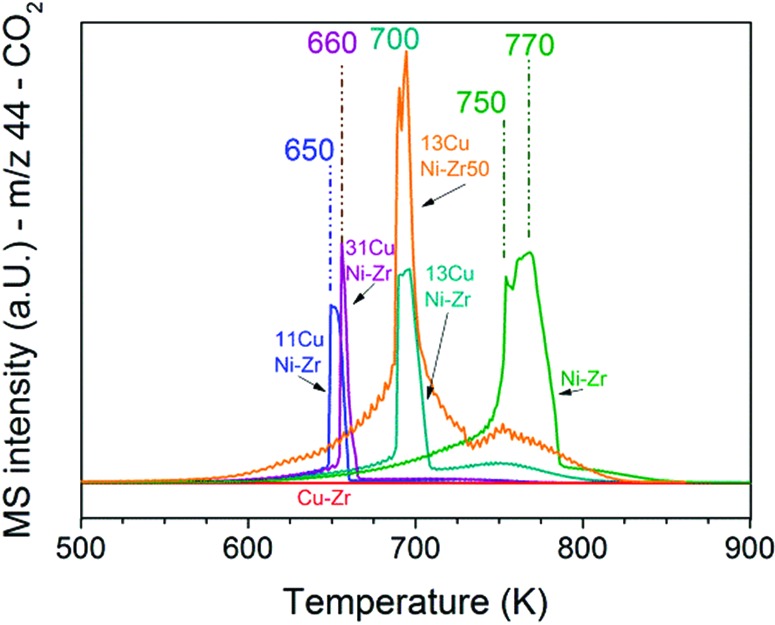
TPO performed after the methane decomposition reaction over Ni and all CuNi catalysts (shown in Fig. 3). The mass spectrometry trace of CO_2_ (*m*/*z* 44) is displayed upon heating in 20 vol% O_2_/Ar at a rate of 5 K min^–1^.

To obtain insights into coke formation, temperature-programmed oxidation (TPO) was performed after reaction in methane. The amount of carbon dioxide formed during TPO provides information about the amount of coke formed during the reaction, and the temperature, which is needed to burn off the carbon species, may be characteristic of the carbon bond strength to the catalyst's surface. Fig. 4 shows the production of carbon dioxide observed during TPO in 20% O_2_ in Ar performed directly after methane decomposition (described in the previous section). Over Ni–ZrO_2_ the highest amount of coke was formed and temperatures of 770 K were needed to oxidize most of it. On the nickel-rich bimetallic catalyst 13CuNi–ZrO_2_ the amount of formed CO_2_ was lower by about 50% compared to that on Ni–ZrO_2_. Furthermore, much lower temperatures of around 700 K were required to remove carbon by oxidation. At about 750 K a second smaller CO_2_ evolution peak was observed in the TPO. Over 11CuNi–ZrO_2_ the amount of coke was reduced to 30% compared to that over Ni–ZrO_2_, and the maximum of CO_2_ formation was detected at 650 K. Over the Cu-rich catalyst 31CuNi–ZrO_2_ the amount of carbon was reduced to only 20% compared to that over Ni–ZrO_2_. Here, one needs to keep in mind the lower activity of the Cu-rich compositions compared to that of Ni–ZrO_2_. The maximum of the CO_2_ formation rate was observed at 660 K. When considering the differences in H_2_ production rate (Table 2) and the CO_2_ peak areas observed in the TPO measurements, the addition of Cu lowered the amount of coke formed. A major effect of Cu was, however, the down-shift of the carbon oxidation temperature. Since hardly any methane decomposition reaction took place over the Cu–ZrO_2_ reference material, no coke was burnt off during TPO over this catalyst.

The TPO curves show large CO_2_ desorption peaks in a narrow temperature range as well as broader peaks of CO_2_ formation over a broad temperature range. The narrow peaks were always accompanied by a small peak of water (*m*/*z* 18) which indicates oxidation of CH_*x*_ species in this temperature range. The much broader peak underneath might originate from other carbonaceous species such as filamentous carbon. The presence of CH_*x*_ species indicates that part of the adsorbed methane dissociated incompletely.

In summary, the highest C and CH_*x*_ oxidation temperature was observed on monometallic nickel at 750–770 K. Since the oxidation temperature decreased on the bimetallic catalysts we conclude that C species are less strongly bonded to the bimetallic surface. A similar trend with the composition was noticed as for the activity onset temperature described in the section about TPMd. The highest temperature required to burn off coke was observed for 13CuNi–ZrO_2_, with a CO_2_ evolution maximum at 700 K, while 11CuNi–ZrO_2_ exhibited the lowest carbon oxidation temperature with a CO_2_ evolution maximum at 650 K.

### *In situ* X-ray photoelectron spectroscopy

In order to learn more about the surface composition, oxidation states and carbonaceous species present under the reaction atmosphere, *in situ* XPS at millibar pressures was employed. The bimetallic Ni-rich 1 : 3 composition which showed the most pronounced increase in methane decomposition activity was investigated further by synchrotron-based *in situ* XPS aiming at understanding the nature of the irreversible surface modification process. To avoid further complication of the (already complex) spectra by charging effects, a catalyst with a higher overall metal loading of 50 wt% was utilized after carefully checking the comparability of this material to the respective 5 wt% sample.

### XP spectra during reduction in H_2_

**Fig. 5:**
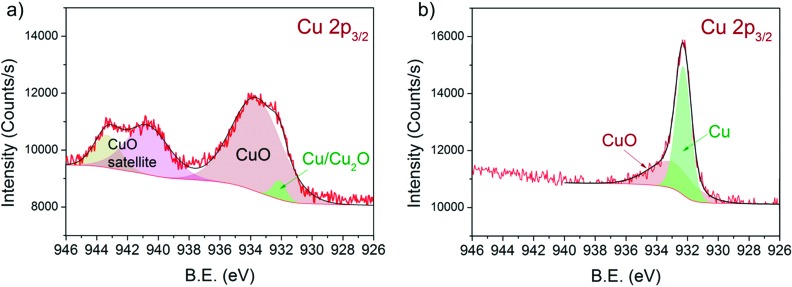
Cu 2p_3/2_ region of 13CuNi–ZrO_2_ 50 in a) 0.10 mbar O_2_ at 573 K and b) 0.25 mbar H_2_ at 673 K for 30 min. The incident photon energy was 1100 eV.

Fig. 5 shows the Cu 2p_3/2_ binding energy region of 13CuNi–ZrO_2_50 during a) oxidation and b) reduction. The peak positions and full width at half-maximum (FWHM) are listed in Table 3.

**Table 3:** Peak positions obtained in XPS measurements on 13CuNi–ZrO_2_50

Species	Peak position (eV)	FWHM (eV)
CuO satellite 2	943.3 ± 0.2	1.9 ± 0.2
CuO satellite 1	940.7 ± 0.2	3.3 ± 0.2
CuO	933.3 ± 0.4	3.7 ± 0.3
New Cu species	933.6 ± 0.2	1.1 ± 0.1
Cu and Cu_2_O	932.3 ± 0.1	0.9 ± 0.1
NiO	854.2 ± 0.2	
Ni	852.6 ± 0.1	
Carbonates/carbon oxygenates	290.1 ± 0.1, 288.8 ± 0.1, 287.8 ± 0.2	1.1 ± 0.2
C–O or C–OH	286.3 ± 0.1	0.9 ± 0.1
C_*x*_H_*y*_ species	285.4 ± 0.1	0.8 ± 0.1
C sp^2^	284.8 ± 0.1	0.6 ± 0.1
Graphitic carbon	284.3 ± 0.1	0.8 ± 0.1
Ni_*x*_C	283.4 ± 0.1	0.9 ± 0.1

The broad peak at 933.3 eV is attributed to CuO^48–53^ with two characteristic shake-up satellite peaks at 940.7 and 943.3 eV. The structure seen in the satellite line is due to the multiplet splitting in the 2p 3d final state.^50^ The small peak located at 932.3 eV is characteristic of Cu_2_O and/or metallic Cu.^48,50,52,53^ During reduction at 673 K the CuO peak decreased in intensity while the signal of reduced Cu species strongly increased. Since the shift of alloyed Cu in CuNi has been reported to be only about 0.2 eV compared to that in monometallic Cu^54–56^ we could not resolve this species.

**Fig. 6:**
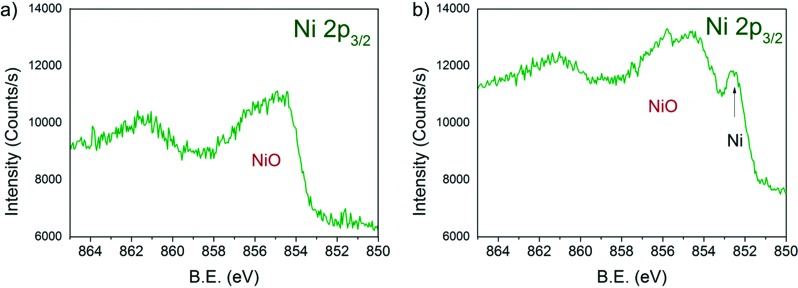
Ni 2p_3/2_ region of 13CuNi–ZrO_2_ 50 in a) 0.10 mbar O_2_ at 573 K and b) 0.25 mbar H_2_ at 673 K for 30 min. The incident photon energy was 1010 eV.

Fig. 6 shows the Ni 2p_3/2_ core levels of 13CuNi–ZrO_2_50 measured during a) oxidation and b) reduction. The peak at 854.3 eV is attributed to the main line of NiO.^23,53,57–59^ In a reducing atmosphere an additional peak appeared at 852.6 eV, which is ascribed to metallic nickel.^53,60,61^ Again, it is not possible to distinguish between nickel interacting with copper and non-interacting with copper due to the small chemical shift of about 0.2 eV^54–56^ which would be expected for Ni alloyed with Cu. In agreement with previously reported findings by IR spectroscopy of CO adsorption on reduced CuNi both oxidized and reduced Ni species are present at the surface after reduction at 673 K, although the bulk consists predominantly of reduced Ni species according to XANES measurements.

Due to the high complexity of the NiO spectrum consisting of shake-up peaks and multiplet states^57,62^ we fitted Ni 2p_3/2_ spectra by the linear combination of reference spectra of NiO and completely reduced Ni. The resulting Ni-to-NiO ratios are summarized in Table 4.

**Table 4:** Proportion of NiO and Ni in Ni 2p_3/2_ spectra fitted by linear combination of reference spectra

Atmosphere and temperature (K)	NiO	Ni
O_2_ – 573	100%	
H_2_ – 573	77 ± 3%	23 ± 3%
CH_4_ – 523	79 ± 3%	21 ± 3%
CH_4_ – 573	80 ± 2%	20 ± 2%
CH_4_ – 623	79 ± 3%	21 ± 3%
CH_4_ – 673	77 ± 3%	23 ± 3%
CH_4_ – 697	75 ± 3%	25 ± 3%
CH_4_ – 723	66 ± 3%	34 ± 3%

Overall, the XP spectra reveal the coexistence of reduced Cu and a mixture of oxidized and metallic Ni species in the near-surface region after reduction.

### XP spectra during methane decomposition

**Fig. 7:**
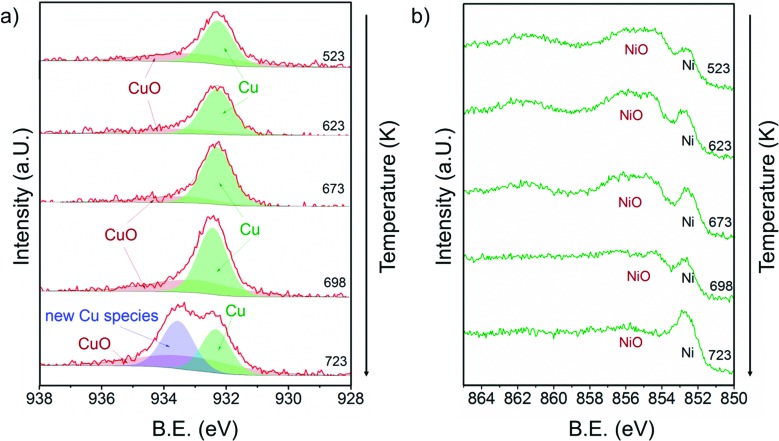
a) Cu 2p_3/2_ and b) Ni 2p_3/2_ core level regions of 13CuNi–ZrO_2_ in 0.25 mbar CH_4_ at rising temperature from 523 to 723 K. The incident photon energies were 1100 eV (Cu) and 1010 eV (Ni).

Fig. 7 shows a) the Cu 2p_3/2_ region and b) the Ni 2p_3/2_ region of 13CuNi–ZrO_2_ during exposure to 0.25 mbar methane at temperatures from 523 to 723 K directly after reduction in H_2_.

At 523 K mainly metallic copper and small amounts of CuO were present in the Cu 2p_3/2_ region. By increasing the temperature the metallic Cu peak became broader and shifted to a slightly higher binding energy indicating further alloy formation between copper and nickel. The amount of nickel oxide decreased with temperature, while the signal of metallic nickel increased. At 723 K a new Cu species at 933.6 eV appeared at the highest temperature. A potential assignment of this species is Cu interacting with surface carbonates;^53^ however, a larger shift to higher binding energies would be expected compared to CuO. CuO core levels have been observed over a broad binding energy range, but in the XP spectrum at 723 K no satellite peaks characteristic of CuO are found, which rather excludes the assignment of the peak at 933.6 eV to CuO. Currently, the nature of this peak remains an open question.

Between 698 and 723 K the biggest changes were observed in the Ni 2p_3/2_ region as well. The amount of NiO decreased significantly, while the concentration of metallic nickel increased in the near-surface region. Note that in the temperature range between 698 and 723 K where the strongest spectral changes occurred the catalytic activity of this catalyst increases dramatically, as shown by TPMd and in agreement with the simultaneously recorded mass spectrometry data.

**Fig. 8:**
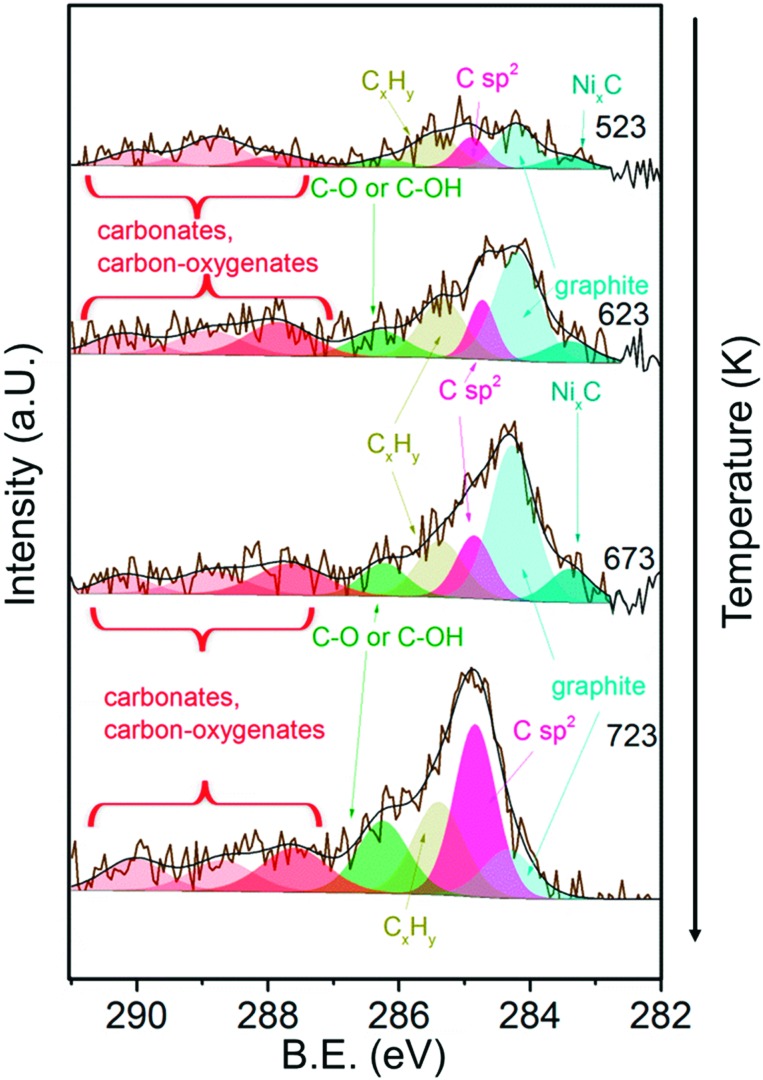
C 1s core level region of 13CuNi–ZrO_2_ in 0.25 mbar CH_4_ at increasing temperature from 523 to 723 K. The incident photon energy was 425 eV.

Fig. 8 shows the C 1s core level region during exposure of 13CuNi–ZrO_2_ to methane at temperatures increasing from 523 to 723 K. A number of different carbon-containing surface species were present in methane. Polycarbonates, carbonates and carbon oxygenates above 287 eV hardly changed with temperature. Various carbonate species were also observed by infrared spectroscopy on ZrO_2_. C–O^63–66^ or C–OH^64,65^ at 286.3 eV increased with temperature, indicating an interaction of carbon with the oxide support or NiO and CuO which had not been reduced up to that temperature. The amount of graphitic carbon^63,67,68^ at 284.3 eV binding energy increased until 673 K but then decreased again at 723 K. The peak at 284.8 eV attributed to C having sp^2^ hybridization^64,68–70^ indicates the formation of carbon nanotubes (CNTs) and increased with increasing temperature. The signal at 285.4 eV corresponds to C_*x*_H_*y*_ species.^66,71^ These species increased upon heating as well. Alternatively, the signal could indicate the presence of structural defects on carbon nanotubes.^70^ Ni_*x*_C^64,65^ appeared at 283.4 eV and increased at first with rising temperature but then completely vanished at 723 K.

In summary, the amount of several carbon species increased during methane decomposition. In particular, the growth of CNTs is significantly enhanced at the highest temperature at which catalytic activity sets in at the same time, while graphitic carbon decreases, either by transformation to CNTs or dissolution in the bulk of the particles.

### Depth profiling

To get more insights into the distribution of Ni, Cu and C species in the surface and subsurface region XP spectra were recorded using different photon energies. The photon energies were varied to obtain photoelectron kinetic energies of 150 eV, 350 eV and 550 eV for each set of spectra of the relevant core level regions (Ni 2p, Cu 2p, C 1s). These depth profiles were performed at 523, 673 and 723 K in methane.

**Fig. 9:**
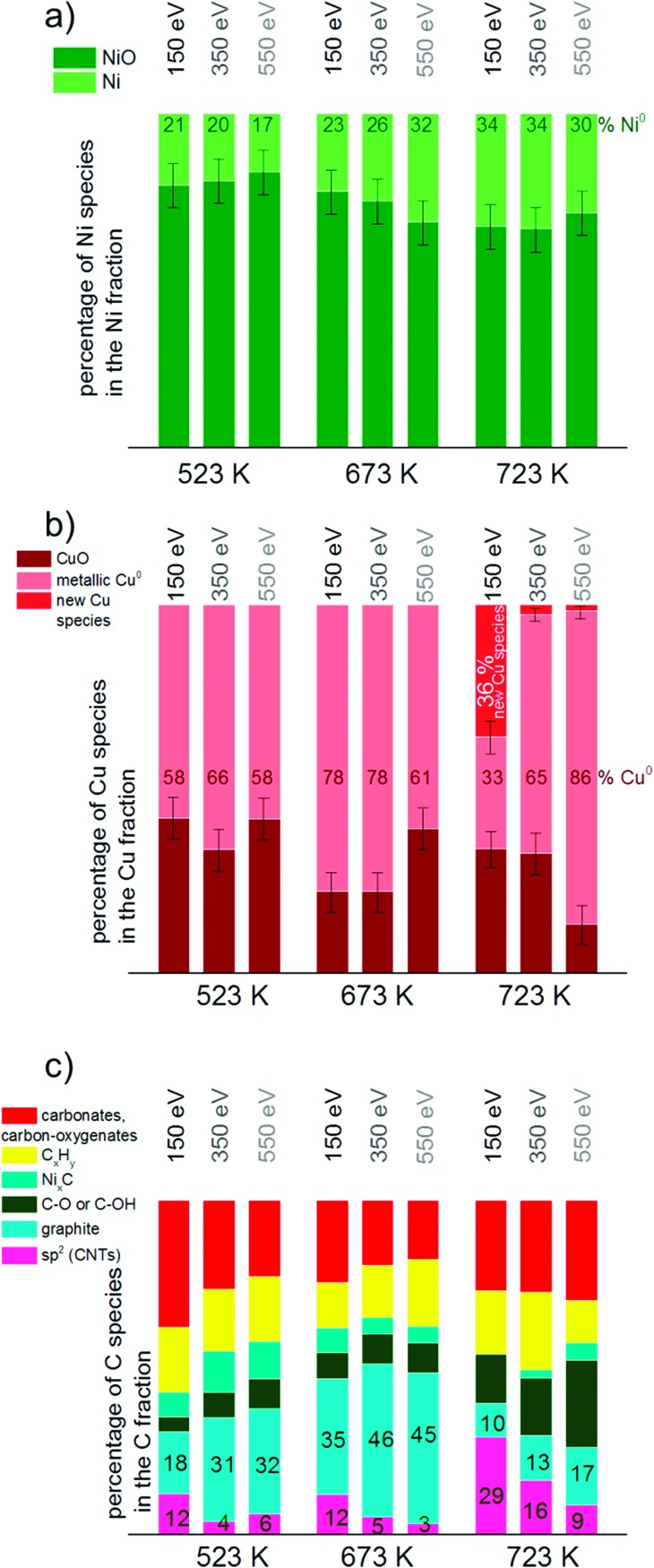
Depth profile of a) Ni, b) Cu and c) C species based on the total amount of Ni, Cu and C, respectively. Spectra in the core level regions Ni 2p_3/2_, Cu 2p_3/2_ and C 1s with kinetic energies of 150, 350 and 550 eV were consulted for the calculations. For clarity of the diagram error bars were left out in c).

Fig. 9 visualizes the relative core level peak areas of the various Cu, Ni and C species based on the respective total areas of Cu, Ni and C signals. Upon heating from 523 to 673 K, the relative amount of metallic Ni increases mainly in the deeper (near-surface) layers. After further heating from 673 to 723 K an increased amount of reduced Ni is present on the surface as well. The amount of metallic Cu increases with temperature. At 723 K the new Cu species appeared which cannot be identified yet (as discussed in the results section). Interestingly, this species is related to the surface of the catalyst and appears only when using photon energies yielding photoelectrons of 150 eV kinetic energies, *i.e.* when measuring with the highest surface sensitivity.

In general, the total amount of carbon species decreases with increasing kinetic energy, indicating that the carbon species are present mostly at the surface of the catalyst. As shown in Fig. 9c), the relative proportion of CNTs increases significantly at 723 K, in particular in the surface-sensitive measurement, as shown by the strongly decreasing amount of sp^2^ hybridized carbon with increasing kinetic energy. Nickel carbide disappeared at 723 K in the most surface-sensitive spectra recorded at 150 eV kinetic energy, while a small amount of carbide is still present in the somewhat deeper near-surface layers. The relative amount of graphitic carbon is highest at 673 K reaction temperature and then decreases considerably at 723 K. This is observed for all photoelectron kinetic energies that were analyzed. The proportion of carbon present as carbonates, carbon-oxygenates and hydrocarbon species does not depend much on reaction temperature and photon energy.

## Discussion

CH_4_ decomposition was studied on bimetallic CuNi–ZrO_2_ catalysts by temperature-programmed reaction, TPO and NAP-XPS in order to get insights into the *in situ* stability of CuNi compounds and the effect of Cu addition on the carbon chemistry. In contrast to monometallic Ni–ZrO_2_, an irreversible activation step was observed in methane at around 700 K, with the exact onset temperature depending on the composition, reduction temperature and metal loading. In the following we will discuss the potential origin and the factors affecting this surface modification process responsible for the onset of catalytic activity and take a closer look at the bimetallic Cu–Ni system.

The question arises as to what is the origin of the observed activation process occurring in methane between 650 and 735 K and leading to an irreversible modification of the catalytic performance of all bimetallic catalysts towards a Ni-like behaviour. Previous work on these materials allowed us to conclude that after reduction the surface of the bimetallic catalysts appears enriched in Cu^25^ as indicated by selective H_2_ chemisorption on Ni and by FTIR spectroscopy of adsorbed CO. Since Cu is practically inactive in methane decomposition a change in surface composition, *e.g.* by Ni segregating to the surface, is a potential reason for the drastic change in CH_4_ decomposition activity. The interaction of Ni with C and/or CH_4_ would be a likely driving force for the proposed surface segregation of Ni.

In contrast to H_2_ chemisorption experiments at room temperature^25^ showing that the surface is enriched in copper after reduction, the surface Cu : Ni ratio observed by XPS at 673 K was nearly similar to the overall Cu : Ni ratio of the catalyst. According to the literature, a copper-enriched top surface layer is in agreement with the lower surface free energy of copper compared to that of nickel.^72^ According to Sachtler *et al.*, the Gibbs free energy of a copper–nickel mixture at 475 K has a maximum at about 30% copper and a minimum at 80% and at 2% copper^73^ in the bulk. Therefore, we expect coexisting phases to be present at lower temperatures. Since the surface energy is lower for Cu compared to that for Ni the Cu-rich alloy phase is likely present at the surface and the Ni-rich alloy phase underneath (core–shell-like structure). For Cu surfaces with Ni monolayers deposited on top it was reported in ref. 74 and 75 that Ni segregates into the first subsurface layer of Cu(100) as well as Cu(111).^76^ By theoretical calculations, Pourovskii *et al.* ^77^ found a rather sharp minimum of the surface energy corresponding to the arrangement of a Ni layer in the second layer of Cu(100). Anyway, this surface composition is only present at low temperature. At elevated temperature the two phases become miscible. According to the Cu–Ni phase diagram, CuNi forms a single-phase alloy above 600 K.

As revealed by XPS measurements under reaction conditions, the increase in activity is clearly accompanied by an increase in the concentration of *metallic* Ni at the surface. Catalytic activity for methane decomposition therefore depends on the alloy surface composition, specifically on the presence of a sufficiently high concentration of metallic Ni at the surface.

Studt *et al.* ^78^ reported Ni segregation to the surface of CuNi alloys due to the higher bond strength towards CO. Li *et al.* ^79^ reported that the dissociative adsorption of methane and the formation of carbon flakes would be a plausible cause for their observation of reconstruction of Ni–Cu metal particles during methane decomposition reaction.^79^ In a remarkable study, Li *et al.* observed a recrystallization of the Ni–Cu alloy on copper–nickel–alumina catalysts during induction of carbon formation in methane decomposition at 773 K.^7^ In a similar way, Ni could be segregated to the very surface layer at elevated temperatures induced by the presence of methane and/or carbon, which consequently leads to an onset of catalytic activity for methane decomposition. Thus, the surface initially enriched in Cu at low temperatures exhibits low methane decomposition activity until the activation step changes the surface concentration of reduced Ni.

Ni segregation to the top surface is a potential explanation for the observed activation step. However, this could not be confirmed by quantitative analysis of the core level spectra, which is complicated by the (very likely) preferential decoration of Ni by carbon species formed over Ni during the reaction of methane. Nevertheless, there is clear evidence by the XPS data that the sudden increase of reactivity can be linked to the higher concentration of reduced Ni at the surface.

When TPMd was carried out after applying a higher reduction temperature of 773 K an activation step was observed as well, although the activation step temperature was lower compared to that for the catalyst reduced at 673 K. Since reduction at 673 K leads to only partially reduced Ni,^25^ the Ni : NiO ratio is increased after reduction at higher temperature. The higher amount of metallic Ni (at or near the surface) after applying a higher reduction temperature could be a likely reason for the down-shift of the activation temperature.

Cu addition to Ni helps in the desired lowering of carbon deposition. TPO measurements showed a reduction of the amount of coke burnt off as CO_2_ by about 50% when comparing Ni and the Ni-rich 13CuNi–ZrO_2_ catalyst exhibiting similar catalytic activity (Table 2). Another effect of the Cu modification, which influences the carbon chemistry and coking behavior, is the decrease in the carbon oxidation temperature, probably due to a weaker bonding of carbon on the CuNi alloy surface.

However, the CuNi system is stable only in a limited range of conditions (temperatures up to around 700 K depending on the composition and pretreatment). Therefore, a key result of this work is the detection of the instability of CuNi under hydrocarbon-rich conditions at higher temperatures (> ~700 K).

## Conclusions

In this work we have investigated the surface chemistry of methane on Ni–ZrO_2_ and bimetallic CuNi–ZrO_2_ catalysts with a focus on the *in situ* stability under relevant reaction conditions, *i.e.* high temperatures and reactive surrounding gas atmosphere methane.

The activity of bimetallic CuNi–ZrO_2_ catalysts for methane decomposition strongly increased after an irreversible activation step occurring at different temperatures depending on the Cu : Ni ratio. By *in situ* synchrotron-based near-ambient pressure (NAP-)XPS we determined the surface composition of the catalysts and the nature of the carbonaceous species present and correlated these data with the catalytic performance. The sudden increase in catalytic activity could be explained by an increase in the concentration of reduced Ni atoms at the catalyst surface in the active state, likely as a consequence of the interaction with methane. XPS showed the appearance of an additional Cu species on the catalyst surface, when catalytic activity sets in. This species could not be conclusively identified up to now, but is related to the catalyst surface according to the depth profiles recorded.

C 1s core level spectra revealed the presence of various carbonaceous species. The signal attributed to carbon nanotubes increased when activity sets in, while the amount of graphite and carbide decreased again. Cu addition to Ni improved the desired resistance against carbon deposition by lowering the amount of coke formed. A lower temperature was required to burn off carbon from the bimetallic catalysts compared to Ni–ZrO_2_.

As a key result on the Cu-modification of Ni catalysts, we conclude that the CuNi alloy shows limited stability under relevant reaction conditions. This system is stable only in a limited range of temperature up to ~700 K in a hydrocarbon atmosphere. Beyond this temperature, the alloy phase becomes unstable, and carbon-induced segregation of Ni species induces a fast increase in methane decomposition rate. In view of the applicability of this system, a detailed understanding of the stability and surface composition of the bimetallic phases present and the influence of the Cu promoter on the surface chemistry under actual reaction conditions are essential.

## References

[cit1] Li B., Kado S., Mukainakano Y., Miyazawa T., Miyao T., Naito S., Okumura K., Kunimori K., Tomishige K. (2007). J. Catal..

[cit2] Matsumura Y., Nakamori T. (2004). Appl. Catal., A.

[cit3] Rostrup-Nielsen J. R., Sehested J., Norskov J. K. (2002). Adv. Catal..

[cit4] Ross J. R. H., van Keulen A. N. J., Hegarty M. E. S., Seshan K. (1996). Catal. Today.

[cit5] Berrocal G. P., Da S. A. L. M., Assaf J. M., Albornoz A., Rangel M. D. C. (2010). Catal. Today.

[cit6] Otsuka K., Ushiyama T., Yamanaka I. (1993). Chem. Lett..

[cit7] Li Y., Chen J., Ma Y., Zhao J., Qin Y., Chang L. (1999). Chem. Commun..

[cit8] Li Y., Chen J., Qin Y., Chang L. (2000). Energy Fuels.

[cit9] Li Y., Chen J., Chang L., Qin Y. (1998). J. Catal..

[cit10] Li Y., Chen J., Chang L. (1997). Appl. Catal., A.

[cit11] Piao L., Li Y., Chen J., Chang L., Lin J. Y. S. (2002). Catal. Today.

[cit12] Zhang X. X., Li Z. Q., Wen G. H., Fung K. K., Chen J., Li Y. (2001). Chem. Phys. Lett..

[cit13] Chen J., Li Y., Li Z., Zhang X. (2004). Appl. Catal., A.

[cit14] Li Y., Chen J., Chang L., Zhao J. (1998). Stud. Surf. Sci. Catal..

[cit15] Li Y., Li D., Wang G. (2011). Catal. Today.

[cit16] Ashok J., Kumar S. N., Subrahmanyam M., Venugopal A. (2008). Catal. Lett..

[cit17] Ermakova M. A., Ermakov D. Y. (2002). Catal. Today.

[cit18] Guil-Lopez R., La Parola V., Pena M. A., Fierro J. L. G. (2012). Int. J. Hydrogen Energy.

[cit19] Li X., Zhang Y., Smith K. J. (2004). Appl. Catal., A.

[cit20] Gavrielatos I., Drakopoulos V., Neophytides S. G. (2008). J. Catal..

[cit21] Molenbroek A. M., Norskov J. K., Clausen B. S. (2001). J. Phys. Chem. B.

[cit22] Rostrup-Nielsen J. R., Alstrup I. (1999). Catal. Today.

[cit23] Triantafyllopoulos N. C., Neophytides S. G. (2006). J. Catal..

[cit24] Li Z., Chen J., Zhang X., Li Y., Fung K. K. (2002). Carbon.

[cit25] Kitla A., Safonova O. V., Foettinger K. (2013). Catal. Lett..

[cit26] Ashok J., Subrahmanyam M., Venugopal A. (2008). Int. J. Hydrogen Energy.

[cit27] Echegoyen Y., Suelves I., Lazaro M. J., Moliner R., Palacios J. M. (2007). J. Power Sources.

[cit28] Lazaro M. J., Echegoyen Y., Suelves I., Palacios J. M., Moliner R. (2007). Appl. Catal., A.

[cit29] Park E. W., Moon H., Park M.-S., Hyun S. H. (2009). Int. J. Hydrogen Energy.

[cit30] Suelves I., Lazaro M. J., Moliner R., Echegoyen Y., Palacios J. M. (2006). Catal. Today.

[cit31] Monzón A., Latorre N., Ubieto T., Royo C., Romeo E., Villacampa J. I., Dussault L., Dupin J. C., Guimon C., Montioux M. (2006). Catal. Today.

[cit32] Cunha A. F., Órfão J. J. M., Figueiredo J. L. (2009). Int. J. Hydrogen Energy.

[cit33] Liu H., Zhang R., Yan R., Li J., Wang B., Xie K. (2012). Appl. Surf. Sci..

[cit34] Fottinger K., van Bokhoven J. A., Nachtegaal M., Rupprechter G. (2011). J. Phys. Chem. Lett..

[cit35] Haghofer A., Foettinger K., Girgsdies F., Teschner D., Knop-Gericke A., Schloegl R., Rupprechter G. (2012). J. Catal..

[cit36] Foettinger K. (2013). Catal. Today.

[cit37] Foettinger K., Rupprechter G. (2014). Acc. Chem. Res..

[cit38] Rameshan C., Stadlmayr W., Penner S., Lorenz H., Memmel N., Haevecker M., Blume R., Teschner D., Rocha T., Zemlyanov D., Knop-Gericke A., Schloegl R., Kloetzer B. (2012). Angew. Chem., Int. Ed..

[cit39] Rameshan C., Stadlmayr W., Weilach C., Penner S., Lorenz H., Haevecker M., Blume R., Rocha T., Teschner D., Knop-Gericke A., Schloegl R., Memmel N., Zemlyanov D., Rupprechter G., Kloetzer B. (2010). Angew. Chem., Int. Ed..

[cit40] Rameshan R. Mayr L. Kloetzer B. Knop-Gericke A. Haevecker M. Blume R. Zemlyanov D. Penner S., Catal. Lett. , submitted . 10.1016/j.jcat.2012.08.008 PMC3485566 23226689

[cit41] Bluhm H., Haevecker M., Knop-Gericke A., Kleimenov E., Schloegl R., Teschner D., Bukhtiyarov V. I., Ogletree D. F., Salmeron M. (2004). J. Phys. Chem. B.

[cit42] Yeh J. J., Lindau I. (1985). At. Data Nucl. Data Tables.

[cit43] Alvarez-Rodriguez J., Cerro-Alarcon M., Guerrero-Ruiz A., Rodriguez-Ramos I., Arcoya A. (2008). Appl. Catal., A.

[cit44] Cangiano M. D. L. A., Ojeda M. W., Carreras A. C., Gonzalez J. A., Ruiz M. D. C. (2010). Mater. Charact..

[cit45] Pérez-Hernández R., Mondragón Galicia G., Mendoza Anaya D., Palacios J., Angeles-Chavez C., Arenas-Alatorre J. (2008). Int. J. Hydrogen Energy.

[cit46] Choudhary T. V., Sivadinarayana C., Chusuei C. C., Klinghoffer A., Goodman D. W. (2001). J. Catal..

[cit47] Ferreira-Aparicio P., Rodríguez-Ramos I., Guerrero-Ruiz A. (1997). Appl. Catal., A.

[cit48] Jones S. D., Neal L. M., Hagelin-Weaver H. E. (2008). Appl. Catal., B.

[cit49] Espinos J. P., Morales J., Barranco A., Caballero A., Holgado J. P., Gonzalez-Elipe A. R. (2002). J. Phys. Chem. B.

[cit50] Ghijsen J., Tjeng L. H., Van E. J., Eskes H., Westerink J., Sawatzky G. A., Czyzyk M. T. (1988). Phys. Rev. B: Condens. Matter Mater. Phys..

[cit51] Liu Z., Amiridis M. D., Chen Y. (2005). J. Phys. Chem. B.

[cit52] Tong W., West A., Cheung K., Yu K.-M., Tsang S. C. E. (2013). ACS Catal..

[cit53] Wagner C. D., Riggs W. M., Davis L. E., Moulder J. F. and Muilenberg G. E., Handbook of X-Ray Photoelectron Spectroscopy, ed., Perkin-Elmer Corporation, Physical Electronics Division, Eden Prairie, Minn. 5534, 1979.

[cit54] Chen L.-C., Lin S. D. (2011). Appl. Catal., B.

[cit55] Naghash A. R., Etsell T. H., Xu S. (2006). Chem. Mater..

[cit56] Koschel H., Held G., Steinruck H. P. (2000). Surf. Sci..

[cit57] Preda I., Gutierrez A., Abbate M., Yubero F., Mendez J., Alvarez L., Soriano L. (2008). Phys. Rev. B: Condens. Matter Mater. Phys..

[cit58] Guczi L., Stefler G., Geszti O., Sajo I., Paszti Z., Tompos A., Schay Z. (2010). Appl. Catal., A.

[cit59] Chia-Ching W., Cheng-Fu Y. (2013). Nanoscale Res. Lett..

[cit60] Powell C. J. (2012). J. Electron Spectrosc. Relat. Phenom..

[cit61] Pawelec B., Damyanova S., Arishtirova K., Fierro J. L. G., Petrov L. (2007). Appl. Catal., A.

[cit62] Grosvenor A. P., Biesinger M. C., Smart R. S. C., McIntyre N. S. (2006). Surf. Sci..

[cit63] Bastl Z. (1995). Collect. Czech. Chem. Commun..

[cit64] Kovács G. J., Bertóti I., Radnóczi G. (2008). Thin Solid Films.

[cit65] Laidani N., Calliari L., Speranza G., Micheli V., Galvanetto E. (1998). Surf. Coat. Technol..

[cit66] Rodriguez N. M., Anderson P. E., Wootsch A., Wild U., Schlögl R., Paál Z. (2001). J. Catal..

[cit67] Evans S., Thomas J. M. (1977). Proc. R. Soc. London, Ser. A.

[cit68] Wilson J. I. B., Walton J. S., Beamson G. (2001). J. Electron Spectrosc. Relat. Phenom..

[cit69] Jackson S. T., Nuzzo R. G. (1995). Appl. Surf. Sci..

[cit70] Savva P. G., Polychronopoulou K., Ryzkov V. A., Efstathiou A. M. (2010). Appl. Catal., B.

[cit71] Belton D. N., Schmieg S. J. (1990). J. Vac. Sci. Technol., A.

[cit72] Zhu L., Depristo A. E. (1997). J. Catal..

[cit73] Sachtler W. M. H., Van D. P. P. (1969). Surf. Sci..

[cit74] Kim S. H., Lee K. S., Min H. G., Seo J., Hong S. C., Rho T. H., Kim J.-S. (1997). Phys. Rev. B: Condens. Matter Mater. Phys..

[cit75] Alkemade P. F. A., Fortuin H., Balkenende R., Habraken F. H. P. M., Van der Weg W. F. (1990). Surf. Sci..

[cit76] Tzeng Y. R., Wu H. T., Shiang D., Tsong T. T. (1993). Phys. Rev. B: Condens. Matter Mater. Phys..

[cit77] Pourovskii L. V., Skorodumova N. V., Vekilov Y. K., Johansson B., Abrikosov I. A. (1999). Surf. Sci..

[cit78] Studt F., Abild-Pedersen F., Wu Q., Jensen A. D., Temel B., Grunwaldt J.-D., Nørskov J. K. (2012). J. Catal..

[cit79] Li D., Chen J., Li Y. (2009). Int. J. Hydrogen Energy.

